# MaxEnt Projections of Climate‐Driven Distribution Shifts for *Daphniphyllum calycinum* in China

**DOI:** 10.1002/ece3.74138

**Published:** 2026-07-31

**Authors:** Yangzhou Xiang, Suhang Li, Qiong Yang, Ying Liu, Bin Yao, Huilin Dong, Yuan Li

**Affiliations:** ^1^ School of Geography and Resources Guizhou Education University Guiyang China; ^2^ School of Biological Sciences Guizhou Education University Guiyang China; ^3^ State Key Laboratory of Tree Genetics and Breeding, Institute of Ecology Conservation and Restoration Chinese Academy of Forestry Beijing China; ^4^ Grasslands and Sustainable Farming, Production Systems Unit Natural Resources Institute Finland Maaninka Finland

**Keywords:** climate change, *Daphniphyllum calycinum*, habitat suitability, medicinal plants, parameter optimization, species distribution models

## Abstract

Understanding how climate change impacts species distribution is crucial for conservation and sustainable resource use. This study projects the distribution shifts of *Daphniphyllum calycinum* Benth., an ecologically and medicinally important tree species in China, under future climate scenarios using an optimized Maximum Entropy (MaxEnt) model. By integrating 323 occurrence records with 10 environmental variables, we applied a rigorous parameter optimization framework using the ENMeval R package to select optimal settings (regularization multiplier = 2.0, feature classes = HPT), thereby reducing overfitting and improving model transferability. The optimized MaxEnt model achieved high predictive accuracy (AUC = 0.959, TSS = 0.8). Annual mean temperature (Bio1, 35.7%), precipitation of the driest month (Bio14, 33.7%), and annual precipitation (Bio12, 23.7%) were identified as the dominant environmental drivers influencing species distribution. Under current climate conditions, the total suitable habitat covers approximately 110.28 × 10^4^ km^2^, primarily in southern China. Future projections under three Shared Socioeconomic Pathway (SSP) scenarios (SSP126, SSP370, SSP585) for the 2050s‐2090s indicate a potential expansion of suitable habitat, with the largest gain under SSP585 (i.e., expanding to 161.64 × 10^4^ km^2^ by the 2050s, representing a 46.6% increase relative to the current area). However, this net expansion is accompanied by localized habitat loss and, under certain scenarios, a declining proportion of highly suitable areas, revealing nuanced climate vulnerability. The distribution centroid is projected to shift northwestward by 96–140 km by the 2050s across scenarios, indicating a range shift toward higher latitudes and altitudes. Despite this overall expansion, localized habitat loss under higher emission scenarios reveals climate vulnerability, underscoring the need for proactive conservation in contraction zones. By integrating optimized model parameters with multi‐scenario future projections, this study provides a robust methodological framework and offers spatially explicit guidance for prioritizing in situ conservation and sustainable cultivation of this valuable species under climate change.

## Graphical abstract

Using an optimized MaxEnt model, this study projects climate‐driven distribution shifts of Daphniphyllum calycinum, an ecologically and medicinally important subtropical tree in China. Results reveal that annual mean temperature and dry season precipitation primarily govern its distribution, with future warming scenarios projecting significant habitat expansion and northwestward range shifts. These findings provide critical spatial guidance for conservation planning and sustainable cultivation of this valuable species under climate change.

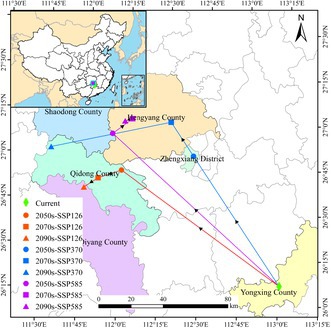

## 1 Introduction

Global climate change is profoundly altering species distributions, population dynamics, and ecosystem structure and function. These changes occur through shifts in temperature, precipitation regimes, and the frequency and intensity of extreme climate events (Pecl et al. 2017; Scheffers and Pecl 2019). Plant species are particularly sensitive to such changes due to their high dependence on specific climatic conditions for survival, growth, and reproduction. Consequently, their distributions serve as key bioindicators of environmental change (Chen et al. 2011; De Frenne et al. 2021).

This sensitivity is especially acute for *Daphniphyllum calycinum* Benth. ( *D. calycinum* ), a species of dual ecological and medicinal importance. Ecologically, it is an important component of subtropical evergreen broadleaf forests in China, contributing to forest biodiversity and community stability (Xia et al. 2018). Medicinally, *D. calycinum* has attracted growing interest due to its structurally diverse alkaloids, which have shown in vitro cytotoxic and anti‐inflammatory activities (Guo et al. 2025; Tang et al. 2024). Although these findings are preliminary (based on cellular assays and not yet validated in vivo), they point to the species' potential as a source of drug leads. Crucially, this emerging medicinal value, together with current reliance on wild‐harvested material, underscores the need to assess climate risks to its natural populations. Such an assessment is a prerequisite for designing sustainable conservation and cultivation strategies. However, wild populations face escalating threats from habitat loss and fragmentation driven by combined pressures of human activities and climate change (Liu et al. 2019; Lu et al. 2020). Although *D. calycinum* is currently assessed as Least Concern on the IUCN Red List (Wu and Qin 2018), its documented medicinal value, increasing market demand, and potential vulnerability to climate change and overharvesting underscore the importance of establishing sustainable supply through domestication and cultivation planning, particularly under future climate uncertainty.

Species distribution models (SDMs) constitute a fundamental methodology in ecology and biogeography, quantifying species‐environment relationships through mathematical functions that integrate known occurrence records with environmental variables to predict potential suitable habitats across spatiotemporal scales (Elith and Leathwick 2009; Franklin 2023). Among these approaches, the Maximum Entropy model (MaxEnt) has gained predominant application due to its robust predictive performance when handling presence‐only data and limited sample sizes (Elith et al. 2011; Phillips et al. 2006). Recent studies have demonstrated its particular utility in guiding medicinal plant resource surveys and introduction area planning (Tang et al. 2021; Wang, Liu, et al. 2023; Yang et al. 2024).

Nevertheless, a prevalent limitation in earlier applications involves excessive reliance on default parameter configurations (Radosavljevic and Anderson 2014), which are rarely optimal across diverse species and environmental contexts. Suboptimal parameter combinations often induce model overfitting or underfitting. This typically occurs with inappropriate settings, such as in the choice of feature classes and regularization multipliers. Such issues may generate misleading predictions that could translate to substantial economic losses if directly applied to inform introduction practices (Muscarella et al. 2014; Warren and Seifert 2011). Consequently, recent methodological advances emphasize systematic parameter optimization, such as conducting grid searches through the R package ENMeval and selecting optimal models using metrics like the corrected Akaike Information Criterion (AICc). This approach enhances the complexity‐prediction accuracy trade‐off and ensures scientifically defensible outcomes (Kass et al. 2021; Shabani et al. 2016). Additionally, multicollinearity among environmental variables represents another critical factor compromising model stability, necessitating rigorous correlation analysis during variable selection procedures (Dormann et al. 2013).

Notwithstanding the growing recognition of the ecological and medicinal importance of *D. calycinum* , substantial gaps persist in understanding its potential geographic distribution. Current research remains largely limited to either descriptive accounts of its current distribution range or predictive approaches based on restricted environmental variables under single climate scenarios (Huang et al. 2018; Li et al. 2025; Wang et al. 2025; Yi et al. 2016). These studies generally overlook critical methodological considerations, particularly model parameter optimization and systematic environmental variable selection. Furthermore, they lack refined quantitative assessments of spatiotemporal changes in suitable habitats, including the identification of stable, lost, and expansion areas, as well as distribution centroid shifts across multiple future climate scenarios and time periods (Bocedi et al. 2021; Duncanson et al. 2023). These limitations significantly constrain our ability to scientifically evaluate the species' conservation status under climate change, delineate priority protection zones, and identify optimal cultivation areas to ensure sustainable medicinal resource supply (Jung et al. 2021; Razgour et al. 2019).

To address these research gaps and advance both conservation and sustainable utilization of *D. calycinum* , this study develops a rigorously parameter‐optimized MaxEnt model to systematically project the potential distribution dynamics exhibited by the species across China. We integrate multi‐source occurrence records with carefully selected multidimensional environmental variables, employing the BCC‐CSM2‐MR climate model under three Shared Socioeconomic Pathways (SSP126, SSP370, SSP585) to simulate distribution patterns from the present period to the 2050s, 2070s, and 2090s. To address these research gaps, this study addresses the following questions: (1) Can systematic parameter optimization improve MaxEnt model performance and reduce overfitting compared to default settings? (2) Which environmental factors primarily govern the distribution of *D. calycinum* , and what are their ecological response thresholds? (3) How will suitable habitats and distribution centroids shift spatiotemporally under different climate scenarios? (4) Where are the persistent and emerging high‐suitability areas that should be prioritized for in situ conservation and sustainable cultivation?

Based on our research objectives, we propose the following three testable hypotheses: (H1) Optimized MaxEnt parameters (ΔAICc ≤ 2) reduce overfitting versus defaults while retaining high accuracy (AUC ≥ 0.9, TSS ≥ 0.8). (H2) Annual mean temperature, driest month precipitation, and annual precipitation primarily constrain distribution, each showing unimodal or saturation responses with clear thresholds. (H3) Under future climate scenarios (SSP126–585, 2050s–2090s), the climatically suitable habitat for *D. calycinum* will expand north‐westward, with the distribution centroid shifting significantly toward higher latitudes. We hypothesize that new high‐suitability areas will emerge primarily at the northern edge of the current range, rather than in entirely disconnected regions.

## 2 Materials and Methods

### 2.1 Data Compilation and Refinement for Species Occurrence

This study compiled a comprehensive distribution dataset for *D. calycinum* by integrating multiple data sources. Occurrence records were obtained from the Chinese Virtual Herbarium (CVH; https://doi.org/10.15468/dl.xmvhfa), the Global Biodiversity Information Facility (GBIF; https://www.gbif.org), China National Knowledge Infrastructure (CNKI; https://www.cnki.net), and Google Scholar (https://scholar.google.com), with data retrieval conducted from September 12 to 19, 2025.

**FIGURE 1:**
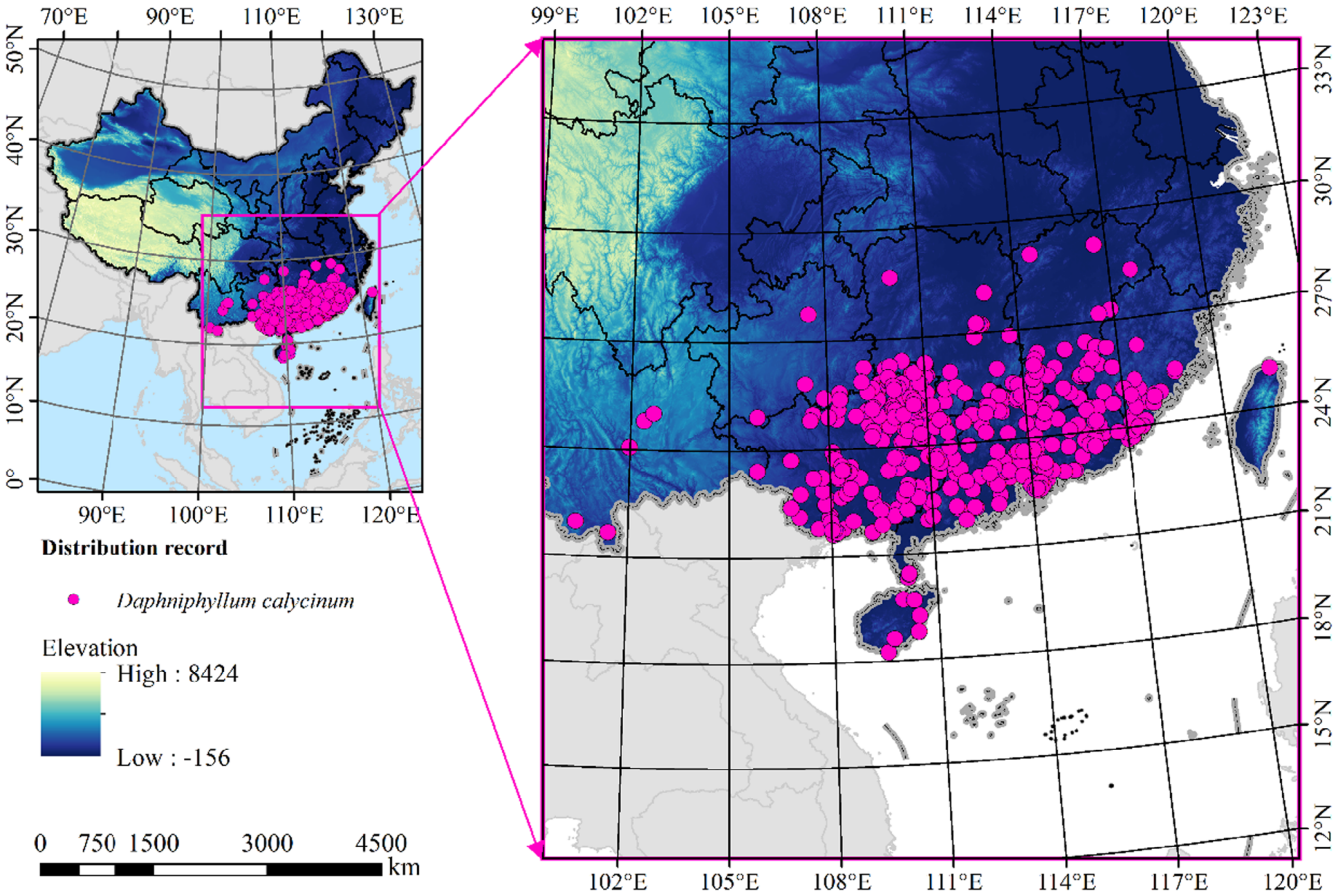
Spatially thinned occurrence records (*n* = 323, 2.5′ grid) of *D. calycinum* used for MaxEnt modeling. Map basemap is from the authorized standard map service of China (Approval No.: GS (2023) 2762).

Initial data processing involved several filtering steps to ensure data quality. First, we retained only records with clear geographic coordinates (latitude and longitude) and excluded those with missing or invalid coordinate values. Second, we manually checked and removed duplicate records based on identical coordinates and source identifiers. Third, we screened all records against known cultivation locations in China (e.g., botanical gardens, experimental farms) using information from field notes and collection descriptions; any record explicitly marked as cultivated or collected from artificial settings was excluded. Fourth, we cross‐referenced records with published distribution maps and expert knowledge to identify and remove potential georeferencing errors (e.g., coordinates falling outside the known range of the species or located in improbable habitats such as urban centers or water bodies). After these filtering steps, 659 unique distribution records remained. To further minimize potential spatial autocorrelation effects on model predictions, we implemented a spatial thinning procedure using ArcGIS 10.8: 2.5′ × 2.5′ grids were established across the study area as spatial thinning units, and following the nearest‐to‐centroid principle, only one representative record was retained per grid cell. This process yielded 323 high‐quality occurrence points (Figure 1), which were stored in CSV format for subsequent modeling. The MaxEnt model (version 3.4.4) was employed (http://biodiversityinformatics.amnh.org/open_source/maxent/; accessed September 20, 2025). All cartographic basemaps of China were sourced from the Standard Map Service of the Ministry of Natural Resources (Approval No.: GS (2023) 2762; http://bzdt.ch.mnr.gov.cn; accessed 22 December 2023), and we strictly adhered to the original boundary representations.

### 2.2 Environmental Variables: Collection and Screening

This study initially selected 24 environmental variables representing four ecological dimensions: climate, topography, vegetation, and anthropogenic influence (Table S1). The selection of these variables was guided by the ecological requirements of *D. calycinum* as a subtropical evergreen broadleaved tree. Climatic variables (Bio1–Bio19) directly regulate plant physiological processes, including growth, reproduction, and survival under temperature and water stress. Topographic variables (elevation, slope, aspect) were included because they influence local microclimate, solar radiation, and soil moisture availability, which in turn affect seedling establishment and adult tree performance. Vegetation productivity, represented by NDVI, serves as a proxy for habitat conditions, including canopy cover and resource availability, which may facilitate or compete with the growth of *D. calycinum* . The Human Footprint Index was included to account for anthropogenic disturbances such as land‐use change and habitat fragmentation, which are known to impact wild populations of medicinal plants in China. The selection comprised: (1) 19 bioclimatic variables and elevation from WorldClim 2.1 (https://www.worldclim.org/data/index.html); (2) topographic parameters (slope and aspect) derived from elevation data using ArcGIS 10.8 surface analysis tools; (3) vegetation characteristics represented by MODIS/Terra Normalized Difference Vegetation Index (NDVI) products (Didan 2015); and (4) anthropogenic pressure quantified through the Human Footprint Index (Mu et al. 2022).

**FIGURE 2:**
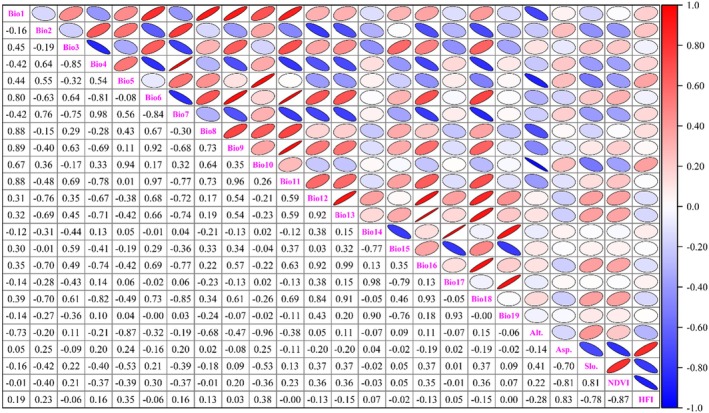
Spearman correlation matrix of the 24 environmental predictors for *D. calycinum* . Alt., asp., and slo. denote altitude, aspect, and slope, respectively.

We addressed multicollinearity as follows: Step 1. Extract environmental values for all 323 occurrence records using the “Extract Multi Values to Points” tool in ArcGIS 10.8. Step 2. Calculate Spearman's rank correlation coefficients for all pairs of the 24 candidate environmental variables using Origin 2025b (Figure 2). Step 3. Retain variables with |*r*| < 0.7 (Dormann et al. 2013). Step 4. For highly correlated pairs (|*r*| ≥ 0.7), compare preliminary model contributions via standalone MaxEnt runs and retain only the variable with higher explanatory power. Step 5. Compile the final variable set for subsequent modeling. The 10 variables retained after this screening process are listed in Table 1. For climate change projections, we utilized the BCC‐CSM2‐MR model (Shi et al. 2020), which demonstrates particular skill in simulating East Asian climate patterns, under three Shared Socioeconomic Pathways (SSP126, SSP370, SSP585) (He et al. 2023) to project distributional changes for the 2050s, 2070s, and 2090s. This model was selected because of its validated performance in simulating regional temperature and precipitation regimes in East Asia. While we acknowledge that using a single climate model introduces uncertainty, our primary focus here is on establishing a methodologically robust MaxEnt framework.

**TABLE 1:** Ten modeling variables retained after multicollinearity screening.

Variable codes	Full names	Units
Bio1	Annual mean temperature	°C
Bio2	Mean diurnal range (Mean of monthly)	°C
Bio5	Max temperature of warmest month	°C
Bio12	Annual precipitation	mm
Bio14	Precipitation of driest month	mm
Bio15	Variation of precipitation seasonality	
Aspect	Aspect	°
Slope	Slope	°
HFI	Human footprint index	
NDVI	Normalized difference vegetation index	

### 2.3 Optimizing Parameters for the MaxEnt Model

To enhance the predictive accuracy and ecological realism of MaxEnt distribution models for *D. calycinum* , we implemented a systematic parameter optimization framework targeting two key determinants of model complexity: feature classes (FC) and regularization multiplier (RM). We optimized MaxEnt model parameters using the ENMeval 2.0.4 package (Kass et al. 2021) in R 4.2.2. A grid search was performed across regularization multipliers (RM) from 0.5 to 4.0 at 0.5 increments and nine feature class (FC) combinations derived from five basic types (linear L, quadratic Q, product P, threshold T, hinge H): L, H, LQ, LQH, LQHP, LQHPT, QHP, QHPT, and HPT (Phillips and Dudík 2008). For each RM–FC combination, we built a MaxEnt model using 323 occurrence records and 10 environmental variables with cross‐validation. Model performance was evaluated using the corrected Akaike Information Criterion (AICc) (Muscarella et al. 2014). The optimal parameter set was defined as the combination with the lowest AICc value; all combinations with ΔAICc ≤ 2 were considered statistically equivalent (Anderson and Gonzalez 2011).

### 2.4 Model Construction and Evaluation

Utilizing 323 georeferenced occurrence records of *D. calycinum* , we employed an optimized MaxEnt framework to model its potential suitable habitats. The modeling protocol incorporated three key steps: first, random partitioning of occurrence data into training (75%) and validation (25%) subsets; second, parameter configuration with cross‐validation, 10,000 background points, a regularization multiplier of 2.0, and HPT feature classes; and third, generation of ensemble habitat suitability through 10 replicate simulations. Model performance was rigorously assessed using the area under the receiver operating characteristic curve (AUC) and true skill statistic (TSS) metrics. Following established conventions, AUC values exceeding 0.9 indicate exceptional predictive accuracy (Phillips and Dudík 2008), while TSS scores above 0.8 represent outstanding model performance (Allouche et al. 2006). The MaxEnt model assumes unlimited dispersal, meaning that all climatically suitable areas are potentially accessible to the species without barriers. It is important to note that our MaxEnt model, like most correlative SDMs, assumes unlimited dispersal, meaning all climatically suitable areas are potentially accessible. Furthermore, it assumes no change in land use or anthropogenic barriers (e.g., the Human Footprint Index was held constant at current levels for future projections). Therefore, our outputs represent the potential climatic suitability for the species, representing an optimistic upper bound, rather than a deterministic forecast of future realized distribution.

### 2.5 Defining Habitat Suitability Classes

We implemented a threshold‐based classification scheme using the 10 percentile training presence criterion (Radosavljevic and Anderson 2014). Based on mean suitability probabilities (P) derived from 10 model replicates, we established four hierarchical suitability categories through raster reclassification in ArcGIS 10.8: unsuitable (*p* < 0.0994), low‐suitability (0.0994 ≤ *p* < 0.3), moderate‐suitability (0.3 ≤ *p* < 0.5), and high‐suitability (*p* ≥ 0.5) habitats (Wang, Xie, et al. 2023). This criterion balances omission and commission errors and is less sensitive to outlier occurrences than the minimum training presence threshold, making it a conservative and widely used choice in species distribution modeling.

### 2.6 Shifting Patterns and Centroids of Suitable Habitats

To elucidate climate‐driven distributional shifts, we conducted comprehensive spatial–temporal analyses incorporating both pattern dynamics and centroid trajectories. Binary habitat distributions were generated by applying the uniform threshold (*p* = 0.0994) to current and future projections across three SSP scenarios (126, 370, 585) and three time periods (2050s, 2070s, 2090s). Spatial overlay techniques identified three distinct transition categories: stable (maintained suitability), lost (suitability degradation), and expansion (suitability emergence) areas (Xiang et al. 2025). Concurrently, distribution centroids were extracted using Zonal Geometry analysis, with Euclidean distance calculations quantifying migration magnitude and directional patterns across temporal gradients (Shi et al. 2024).

## 3 Results

### 3.1 Optimizing Model and Evaluating Accuracy

**FIGURE 3:**
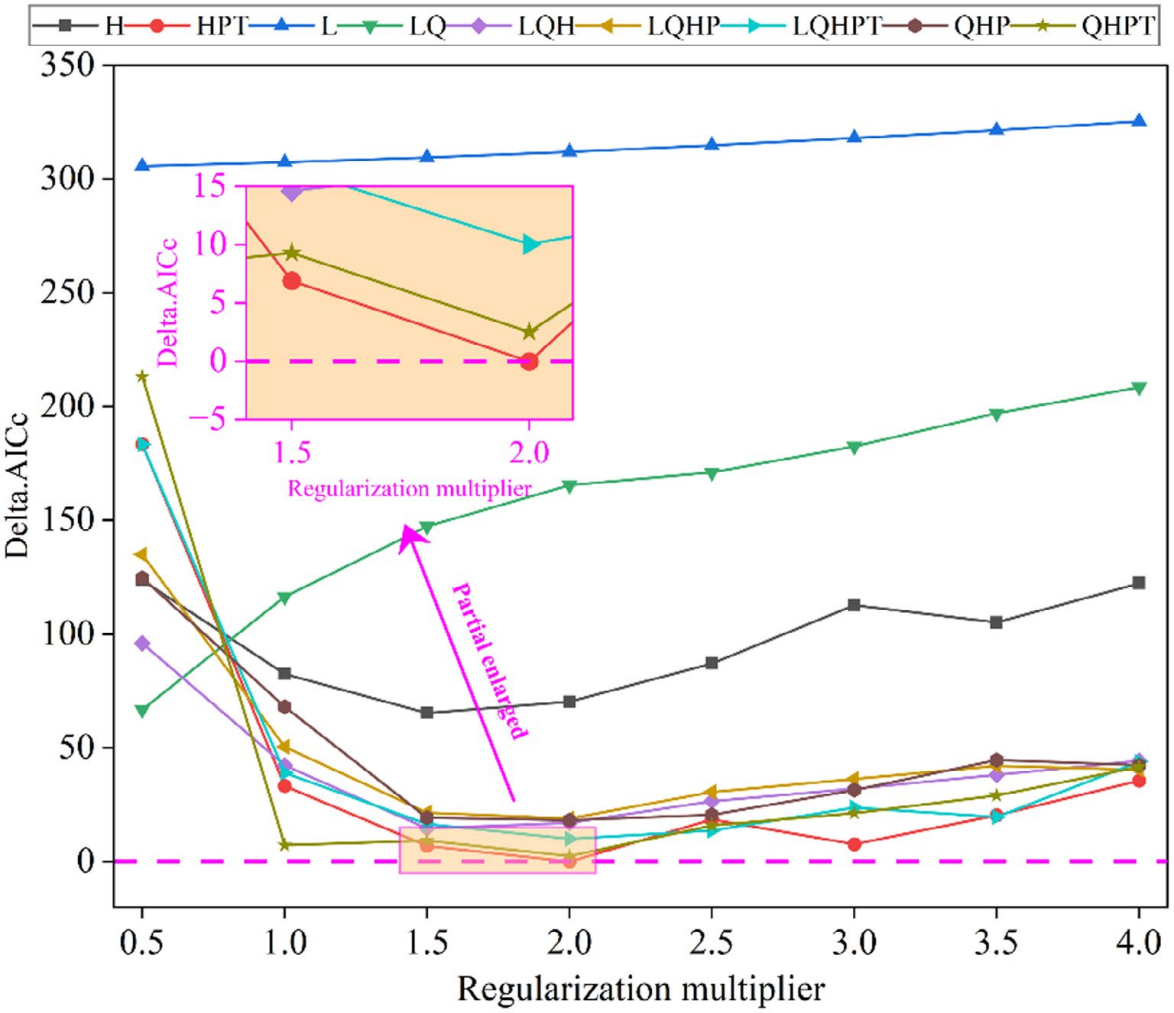
The distribution of delta. AICc values for five feature combinations (L: Linear, Q: Quadratic, H: Hinge, P: Product, T: Threshold) in *D. calycinum* . A pink dashed line denotes the zero‐reference level for comparative purposes.

**FIGURE 4:**
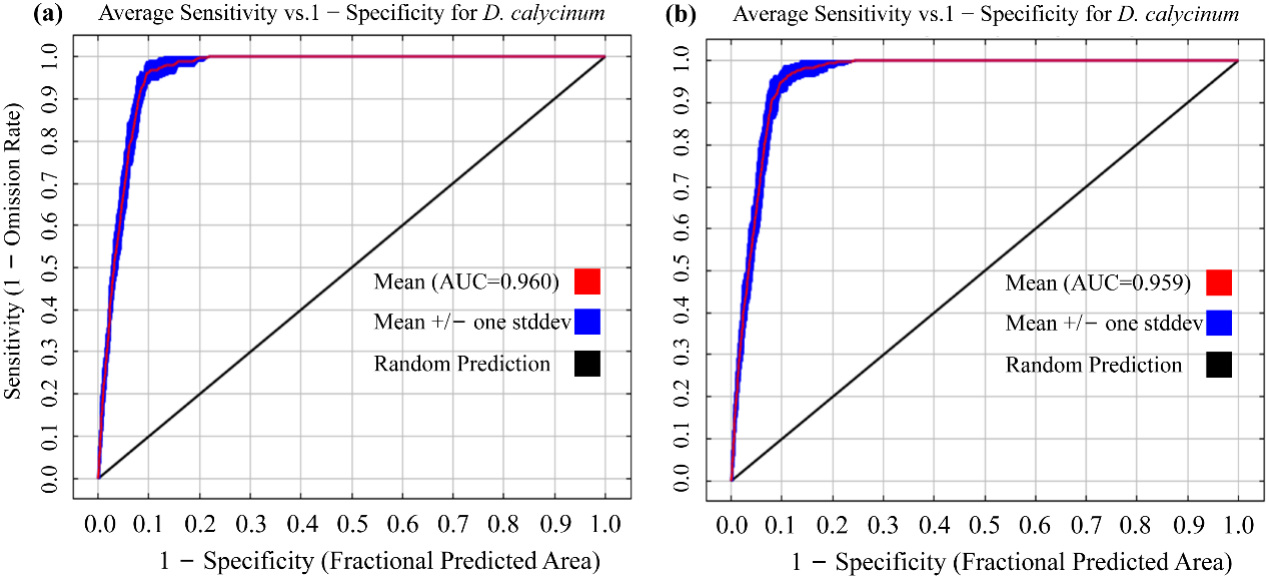
ROC curves of the model with default parameters (a) and with optimized parameters (b).

When using default parameters (RM = 1, FC = LQHP), the model exhibited a Delta.AICc value of 50.42 (Figure 3), substantially exceeding the threshold of ΔAICc ≤ 2 used to identify statistically equivalent models (Anderson and Gonzalez 2011). In contrast, the optimized model (RM = 2.0, FC = HPT) achieved a Delta.AICc of 0. Comparative analysis revealed that both default (AUC = 0.960; Figure 4a) and optimized (AUC = 0.959; Figure 4b) parameters exceeded the threshold for excellent predictive performance (AUC ≥ 0.9) based on 10 replicated cross‐validations. Although the optimized model showed a marginal decrease in AUC (ΔAUC = −0.001), a difference that is not statistically or practically significant, the enhanced regularization strength and simplified feature combination effectively controlled overfitting while maintaining strong explanatory power for species‐environment relationships. Furthermore, the optimized model achieved a TSS value of 0.8, confirming its robust predictive capability.

### 3.2 Key Environmental Factors Shaping the Distribution of *D. calycinum*

**FIGURE 5:**
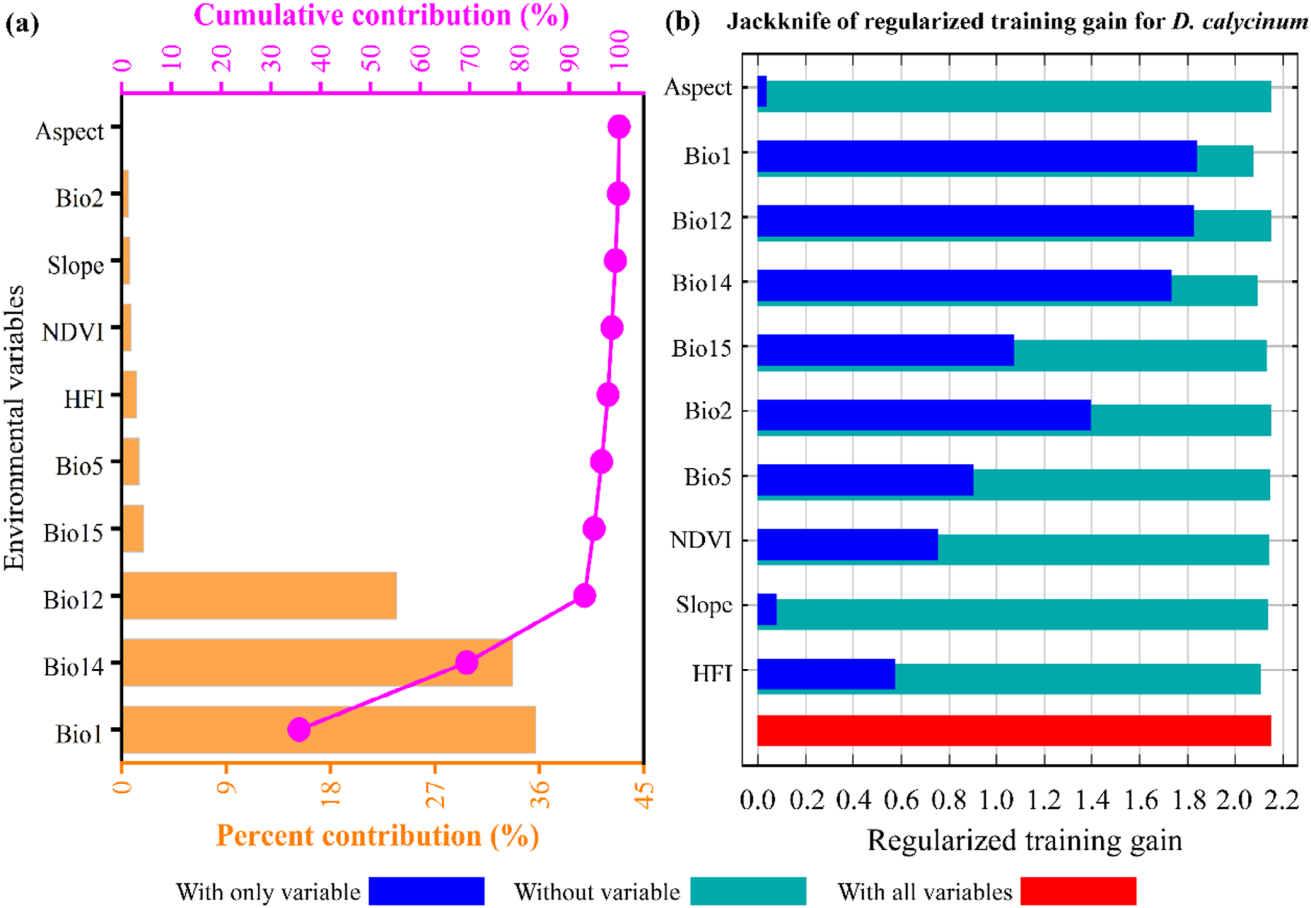
Relative importance of environmental predictors for *D. calycinum* in the MaxEnt model, evaluated by (a) percent contribution and (b) the jackknife test of regularized training gain.

Employing our optimized MaxEnt framework, we conducted a comprehensive assessment of 10 environmental variables governing the distribution of *D. calycinum* , integrating permutation importance analysis and jackknife validation. Variable contributions decreased in the following order: annual mean temperature (Bio1, 35.7%), precipitation of driest month (Bio14, 33.7%), annual precipitation (Bio12, 23.7%), precipitation seasonality (Bio15, 1.9%), maximum temperature of warmest month (Bio5, 1.5%), human footprint index (1.3%), normalized difference vegetation index (0.8%), slope (0.7%), mean diurnal range (Bio2, 0.6%), and aspect (0.1%). Climatic factors collectively dominated the explanatory power (97.1%), substantially surpassing anthropogenic (1.3%), topographic (0.8%), and vegetation (0.8%) influences. Jackknife tests based on regularized training gain (Figure 5b) confirmed the predominant role of climatic variables, with Bio1, Bio12, Bio14, Bio2, and Bio15 achieving superior performance when used independently. Following the established threshold of cumulative contribution rate exceeding 85% (Yang et al. 2022), the three key bioclimatic variables Bio1, Bio14, and Bio12 collectively explained 93.1% of the distributional variance, establishing them as the primary determinants of the species' geographic range in China.

**FIGURE 6:**
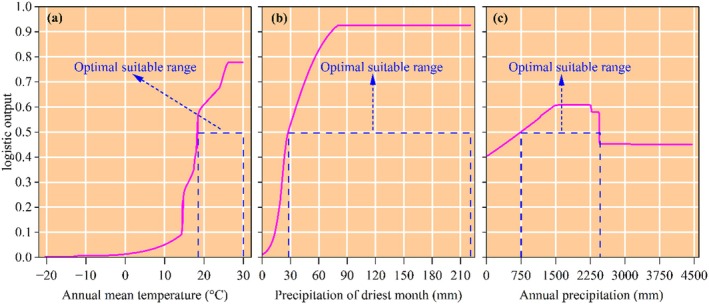
Response curves of *D. calycinum* to three key environmental variables. The curves show the mean response over 10 replicate MaxEnt runs (purple line), and the blue dashed lines indicate the optimal suitability range. (a) Response curve of the annual mean temperature (°C), (b) Response curve of precipitation of driest month (mm), and (c) Response curve of the annual precipitation (mm).

Response curve analysis (Figure 6) revealed distinct ecological thresholds for these critical factors. For annual mean temperature (Bio1), occurrence probability demonstrated a triphasic response: gradual increase from 0.003 to 0.09 across −20.33°C to 14.38°C, rapid ascent to maximum probability (0.78) between 14.39°C and 26.17°C, and subsequent stabilization at higher temperatures (Figure 6a). Precipitation of driest month (Bio14) exhibited a saturation response, with probability surging from 0.01 to 0.93 across 0–80.19 mm before plateauing (Figure 6b). Annual precipitation (Bio12) displayed a unimodal relationship characterized by initial increase (0.40–0.61) across 0–1491.03 mm, stability through 1491.04–2265.81 mm, and sharp decline to 0.45 beyond 2265.82 mm (Figure 6c).

### 3.3 Projected Current Distribution of *D. calycinum*

**FIGURE 7:**
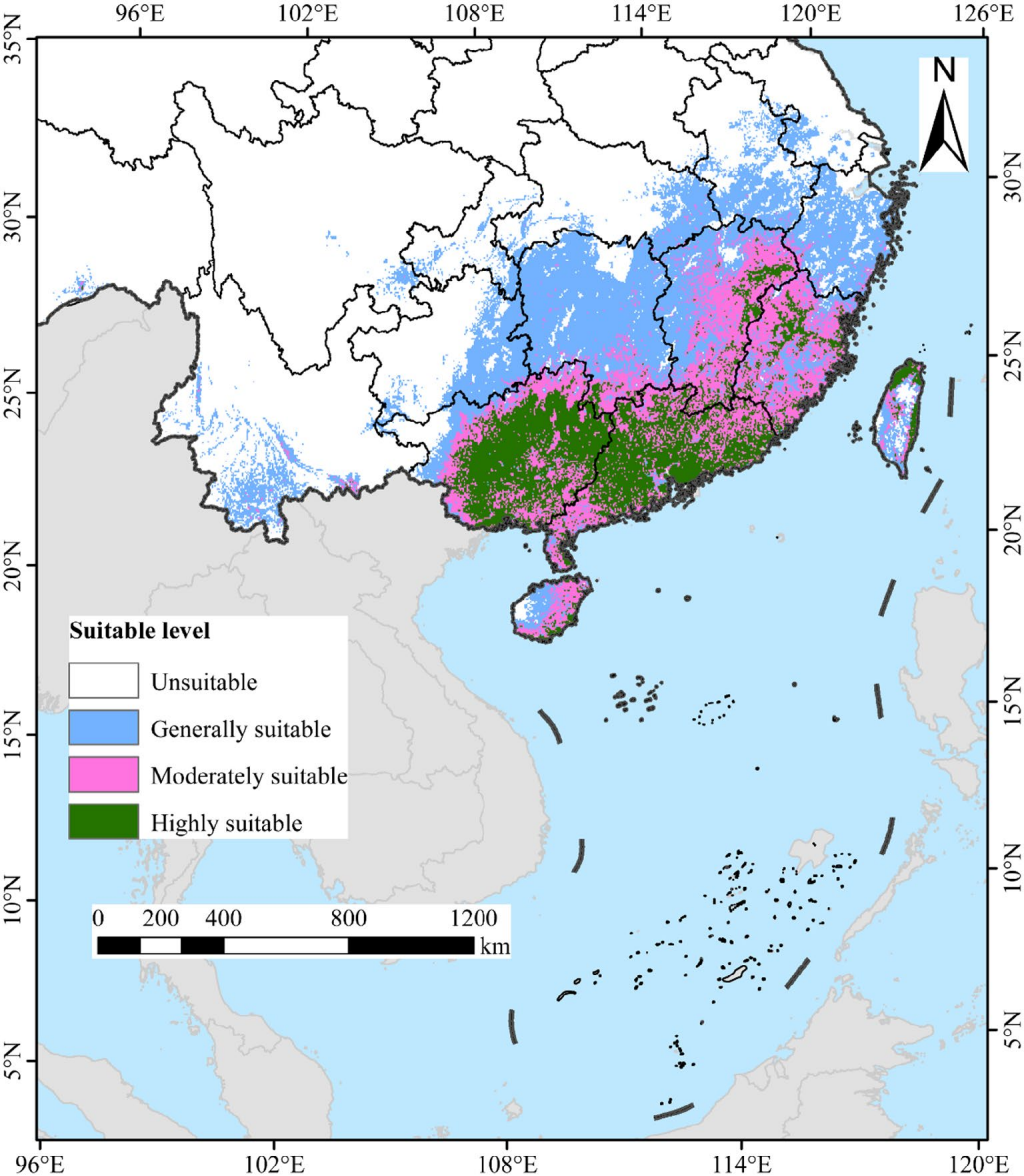
Predicted suitable habitats for *D. calycinum* in China under contemporary climate.

Under current climate scenarios, *D. calycinum* demonstrates a distinct distribution pattern across China (Figure 7). The optimized model predicts a total suitable area of approximately 110.28 × 10^4^ km^2^ in China, accounting for 11.49% of the national territory. High‐suitability areas cover approximately 24.51 × 10^4^ km^2^ (2.55%), mainly distributed in Guangxi Zhuang Autonomous Region, Guangdong Province, northern and eastern Taiwan Province, coastal areas of Fujian Province, and eastern Jiangxi Province. Moderate‐suitability areas encompass approximately 30.00 × 10^4^ km^2^ (3.13%), concentrated in central Jiangxi Province, western Fujian Province, northern and western Guangxi Zhuang Autonomous Region, the mountainous areas of northern Guangdong Province, eastern and southern Hainan Province, and central Taiwan Province. Low‐suitability areas span approximately 55.77 × 10^4^ km^2^ (5.81%), displaying a fragmented distribution pattern across Hunan Province, Zhejiang Province, eastern Guizhou Province, southern Yunnan Province, southeastern Sichuan Province, eastern Hubei Province, central and southern Anhui Province, western Jiangxi Province, western Hainan Province, and southern Taiwan Province.

### 3.4 Potential Distribution Shifts of *D. calycinum* Under Future Climate Change

**FIGURE 8:**
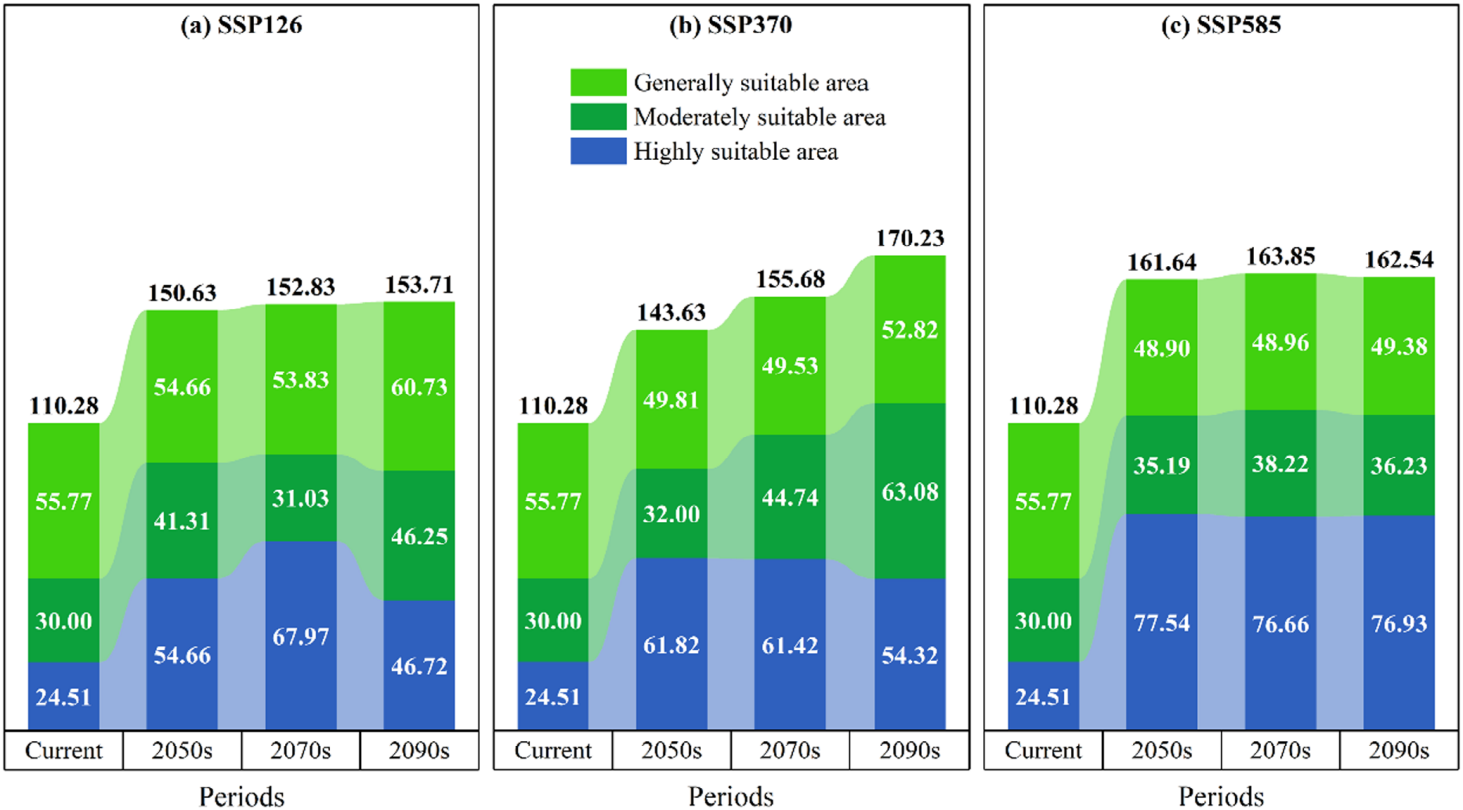
Projected changes in suitable habitat area for *D. calycinum* from current conditions to the 2090s across climate change scenarios (unit: 10^4^ km^2^).

**FIGURE 9:**
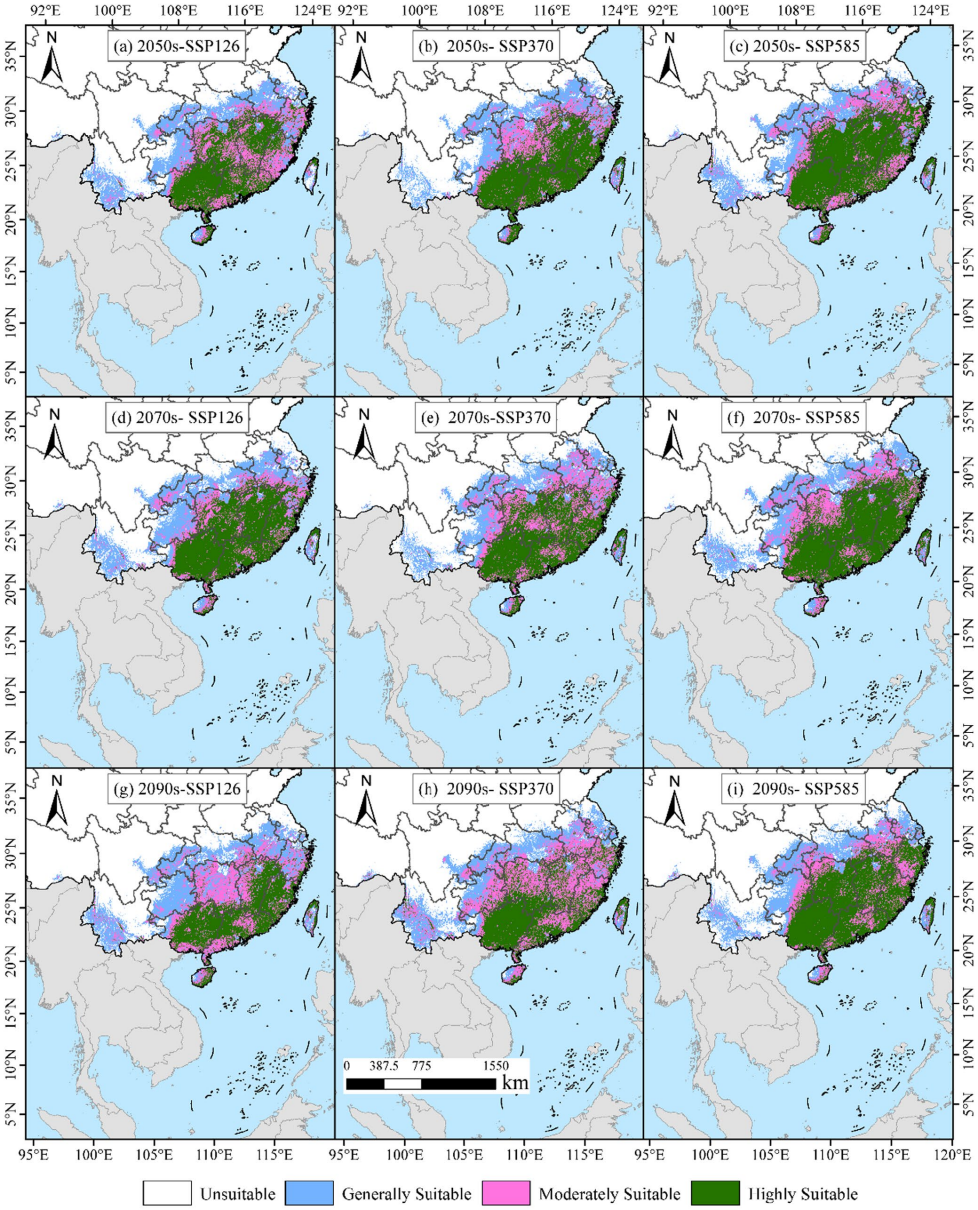
Potentially suitable areas for *D. calycinum* in China under different climate scenarios over multiple periods.

Under the SSP126 climate scenario, *D. calycinum* exhibits sustained expansion in suitable habitat (Figure 8a). By the 2050s, the total suitable area is projected to reach 150.63 × 10^4^ km^2^ (15.69% of China's terrestrial area), comprising 54.66 × 10^4^ km^2^ of low‐suitability, 41.31 × 10^4^ km^2^ of moderate‐suitability, and 54.66 × 10^4^ km^2^ of high‐suitability habitats. High‐suitability areas are primarily distributed across Guangxi Zhuang Autonomous Region, Guangdong Province, Jiangxi Province, along with northern Taiwan Province, northwestern Fujian Province, central Hunan Province, and eastern Hainan Province (Figure 9a). During the 2070s, the total suitable area increases to 152.83 × 10^4^ km^2^ (15.92%), comprising 53.83 × 10^4^ km^2^ (low), 31.03 × 10^4^ km^2^ (moderate), and 67.97 × 10^4^ km^2^ (high). High‐suitability zones expand to be widely distributed across Fujian Province, southwestern Zhejiang Province, and central and southern Hunan Province (Figure 9d). By the 2090s, the total suitable area reaches 153.71 × 10^4^ km^2^ (16.01%), comprising 60.73 × 10^4^ km^2^ (low), 46.25 × 10^4^ km^2^ (moderate), and 46.72 × 10^4^ km^2^ (high), with high‐suitability habitats distributed across Guangxi Zhuang Autonomous Region, central and northern Guangdong Province, and coastal areas of Fujian Province (Figure 9g).

Under the SSP370 scenario, habitat suitability demonstrates fluctuating growth patterns (Figure 8b). In the 2050s, the total suitable area covers 143.63 × 10^4^ km^2^ (14.96%), comprising 49.81 × 10^4^ km^2^ (low), 32.00 × 10^4^ km^2^ (moderate), and 61.82 × 10^4^ km^2^ (high). High‐suitability habitats concentrate predominantly in southern coastal provinces, including Guangxi Zhuang Autonomous Region, Guangdong Province, Jiangxi Province, and Fujian Province (Figure 9b). By the 2070s, the total area expands to 155.68 × 10^4^ km^2^ (16.22%), comprising 49.53 × 10^4^ km^2^ (low), 44.74 × 10^4^ km^2^ (moderate), and 61.42 × 10^4^ km^2^ (high), with high‐suitability ranges remaining relatively stable (Figure 9e). During the 2090s, the total suitable area further increases to 170.23 × 10^4^ km^2^ (17.73%), comprising 52.82 × 10^4^ km^2^ (low), 63.08 × 10^4^ km^2^ (moderate), and 54.32 × 10^4^ km^2^ (high), as high‐suitability zones extend inland to incorporate southern Jiangxi Province and southern Hunan Province (Figure 9h).

The SSP585 scenario maintains the highest overall habitat suitability levels (Figure 8c). By the 2050s, the total suitable area reaches 161.64 × 10^4^ km^2^ (16.84%), comprising 48.90 × 10^4^ km^2^ (low), 35.19 × 10^4^ km^2^ (moderate), and 77.54 × 10^4^ km^2^ (high). High‐suitability habitats are extensively distributed across regions south of the Yangtze River, including Guangxi Zhuang Autonomous Region, Guangdong Province, Jiangxi Province, Hunan Province, western Fujian Province, central Zhejiang Province, northern Taiwan Province, and eastern Hainan Province (Figure 9c). During the 2070s, the total area slightly increases to 163.85 × 10^4^ km^2^ (17.07%), comprising 48.96 × 10^4^ km^2^ (low), 38.22 × 10^4^ km^2^ (moderate), and 76.66 × 10^4^ km^2^ (high). High‐suitability zones expand northward to incorporate southern Anhui Province (Figure 9f). By the 2090s, the total area slightly decreases to 162.54 × 10^4^ km^2^ (16.93%), comprising 49.38 × 10^4^ km^2^ (low), 36.23 × 10^4^ km^2^ (moderate), and 76.93 × 10^4^ km^2^ (high), while high‐suitability habitats continue to maintain extensive coverage across core southern China regions (Figure 9i).

To quantitatively evaluate the changes in habitat suitability under different emission scenarios, we calculated the relative change rates of suitable habitat areas (compared to the current baseline) and the proportion of highly suitable area for each scenario and period (Table 2). Under the low‐emission SSP126 scenario, the total suitable area (TSA) expands gradually (+36.59% to +39.38%), while the proportion of highly suitable area (HSA) within TSA declines from 44.47% (2070s) to 30.39% (2090s), indicating a long‐term structural shift toward lower‐suitability categories. Under the medium‐to‐high emission SSP370 scenario, TSA reaches a maximum expansion of +54.36% by the 2090s; however, the generally suitable area (GSA) contracts (from −10.69% to −5.29%), and the proportion of HSA declines from 43.04% to 31.91%, reflecting gradual habitat quality degradation. Under the high‐emission SSP585 scenario, TSA and HSA show the most pronounced expansions (+46.57% to +48.58% and +212.75% to +216.34%, respectively). In contrast, the generally suitable area (GSA) contracts across all periods (−12.32% to −11.46%), indicating a continued loss of marginal habitat even under the highest emission scenario. Notably, the proportion of HSA remains consistently high (46.79%–47.97%), far exceeding the current baseline (22.23%). Together, these results demonstrate that higher emission intensity not only drives greater total range expansion but also sustains a larger fraction of high‐quality habitats.

**TABLE 2:** Current and future suitable habitat areas for *D. calycinum* under different SSP scenarios (absolute and relative change).

Scenarios‐periods	TSA change (%)	HSA change (%)	Proportion of HSA (%)	MSA change (%)	GSA change (%)
Current baseline			22.23		
SSP126‐2050s	36.59	122.99	36.29	37.72	−1.99
SSP126‐2070s	38.59	177.29	44.47	3.45	−3.48
SSP126‐2090s	39.38	90.60	30.39	54.19	8.89
SSP370‐2050s	30.24	152.21	43.04	6.68	−10.69
SSP370‐2070s	41.17	150.57	39.45	49.15	−11.19
SSP370‐2090s	54.36	121.61	31.91	110.29	−5.29
SSP585‐2050s	46.57	216.34	47.97	17.31	−12.32
SSP585‐2070s	48.58	212.75	46.79	27.42	−12.21
SSP585‐2090s	47.39	213.85	47.33	20.78	−11.46

*Note:* Relative change rate = (*A*_future_ − *A*_current_)/*A*_current_ × 100%, where *A*_future_ and *A*_current_ represent suitable areas under future and current climate, respectively. Positive values indicate expansion, and negative values indicate contraction relative to the current baseline. The proportion of high‐suitability area is calculated as (high‐suitability area/total suitable area) × 100%.

Abbreviations: GSA, generally suitable area; HSA, highly suitable area; MSA, moderately suitable area; TSA, total suitable area.

### 3.5 Changes in Habitat Suitability in Response to Future Climate Scenarios

**FIGURE 10:**
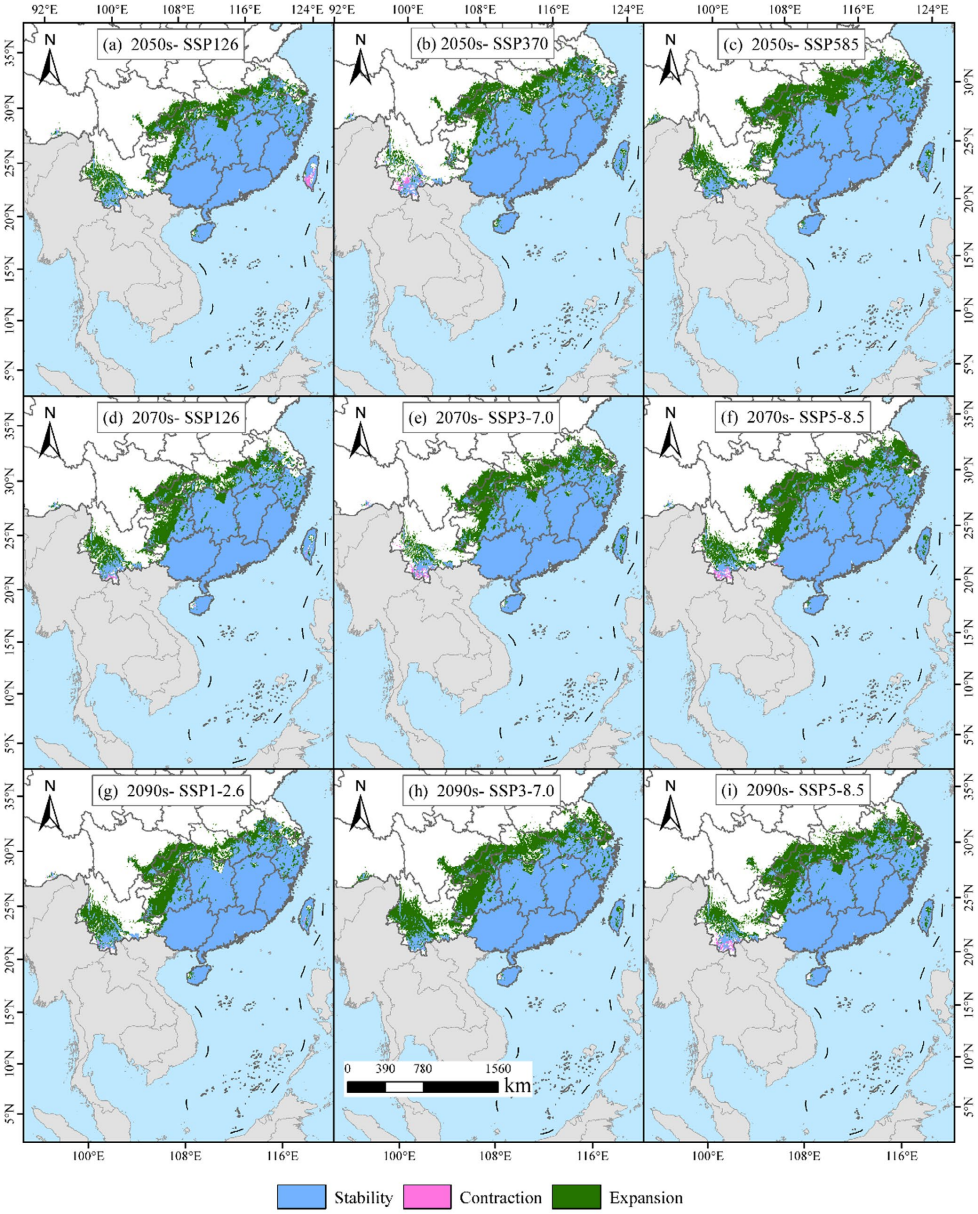
Projected changes in suitable areas for *D. calycinum* under future climate scenarios compared to the present.

Under the SSP126 scenario (Table 3; Figure 10a,d,g), the stable suitable habitat area for *D. calycinum* increased marginally from 134.18 × 10^4^ km^2^ in the 2050s to 134.61 × 10^4^ km^2^ by the 2090s. Over the same period, its proportional retention rate declined from 79.98% to 76.93%. This slight increase in stable area, accompanied by a moderate drop in retention rate, points to a limited but clear expansion of the total potential range. Concurrently, habitat loss area decreased from 0.83 × 10^4^ km^2^ to 0.40 × 10^4^ km^2^, with the loss rate falling from 0.49% to 0.23%. In contrast, expansion area grew steadily from 32.77 × 10^4^ km^2^ to 39.97 × 10^4^ km^2^, with the expansion rate rising from 19.53% to 22.84%.

**TABLE 3:** Projected changes in suitable habitat area for *D. calycinum* across various scenarios.

Scenarios‐periods	Area (10^4^ km^2^)			Rate of change (%)		
	Stability	Contraction	Expansion	Stability	Contraction	Expansion
SSP126‐2050s	134.18	0.83	32.77	79.98	0.49	19.53
SSP126‐2070s	134.60	0.41	28.62	82.26	0.25	17.49
SSP126‐2090s	134.61	0.40	39.97	76.93	0.23	22.84
SSP370‐2050s	134.52	0.49	36.62	78.38	0.28	21.34
SSP370‐2070s	134.84	0.15	54.87	71.02	0.08	28.90
SSP370‐2090s	134.72	0.28	59.02	69.44	0.14	30.42
SSP585‐2050s	134.62	0.38	47.85	73.62	0.21	26.17
SSP585‐2070s	134.72	0.28	57.19	70.10	0.15	29.76
SSP585‐2090s	134.80	0.20	59.64	69.25	0.10	30.64

Under the SSP370 scenario (Table 3; Figure 10b,e,h), the stable habitat area increased slightly from 134.52 × 10^4^ km^2^ (78.38% retention) in the 2050s to 134.72 × 10^4^ km^2^ (69.44% retention) by the 2090s. Despite the near‐stability of the stable area in absolute terms, the retention rate fell sharply by nearly nine percentage points. This decoupling strongly indicates a rapidly expanding total potential range, which is a key signature of climate‐driven northward migration. Habitat loss area decreased from 0.49 × 10^4^ km^2^ to 0.28 × 10^4^ km^2^, with the loss rate dropping from 0.28% to 0.14%. Meanwhile, expansion area more than doubled from 36.62 × 10^4^ km^2^ to 59.02 × 10^4^ km^2^, with the expansion rate rising from 21.34% to 30.42%.

The SSP585 scenario (Table 3; Figure 10c,f,i) revealed more complex dynamics. The stable habitat area initially increased from 134.62 × 10^4^ km^2^ (73.62% retention) in the 2050s to 134.72 × 10^4^ km^2^ (70.10% retention) by the 2070s, before stabilizing at 134.80 × 10^4^ km^2^ (69.25% retention) in the 2090s. Throughout this period, retention rate declined steadily, indicating sustained range expansion despite the near‐stable absolute stable area. Habitat loss area contracted from 0.38 × 10^4^ km^2^ (0.21%) to 0.28 × 10^4^ km^2^ (0.15%), with a further marginal reduction to 0.20 × 10^4^ km^2^ (0.10%) by the 2090s. Expansion area rose from 47.85 × 10^4^ km^2^ to 57.19 × 10^4^ km^2^ (26.17% to 29.76%), and then edged up further to 59.64 × 10^4^ km^2^ (30.64%).

### 3.6 Centroid Shifts of *D. calycinum* Under Future Climate Scenarios

**FIGURE 11:**
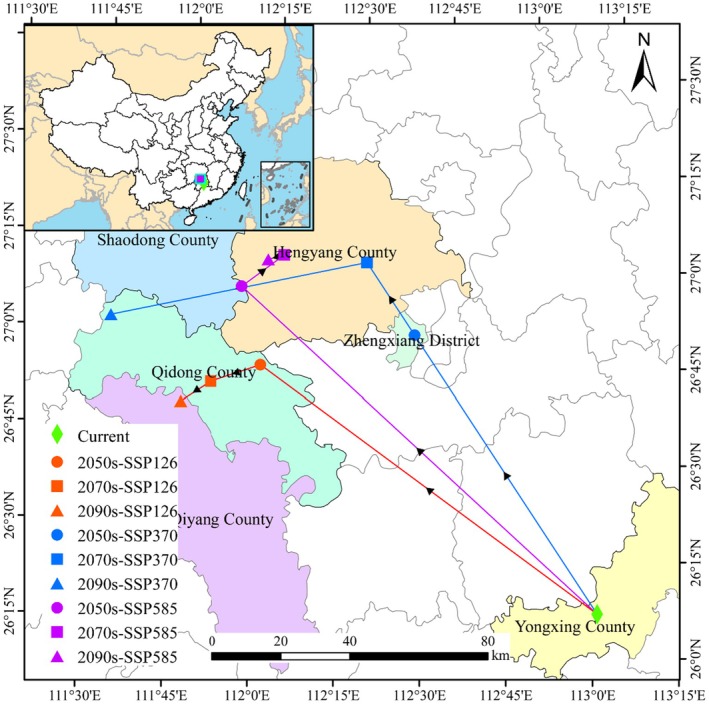
Modeled shifts of distribution centroids for *D. calycinum* from present to the 2090s under SSP126 (red), SSP370 (blue), and SSP585 (pink) scenarios.

Under current climate conditions, the distribution centroid of *D. calycinum* is located in Bianjiang Town, Yongxing County, Chenzhou City, Hunan Province (113°02′ E, 26°08′ N; Figure 11). Under different climate scenarios, the centroid exhibits distinct migration trajectories with the following patterns: (1) Under the SSP126 scenario, by the 2050s, the centroid shifts approximately 121.36 km to the northwest, reaching Hongqiao Subdistrict, Qidong County, Hengyang City (112°06′ E, 26°51′ N). By the 2070s, it continues to migrate approximately 15.26 km to the southwest, arriving at Fengshiyan Town, Qidong County (110°57′ E, 26°49′ N). By the 2090s, it moves further approximately 10.51 km to the southwest, ultimately settling in Wenfushi Town, Qiyang City, Yongzhou City (111°52′ E, 26°46′ N). (2) Under the SSP370 scenario, by the 2050s, the centroid shifts approximately 96.49 km to the northwest, arriving at Huaxing Subdistrict, Zhengxiang District, Hengyang City (112°34′ E, 26°54′ N). By the 2070s, it migrates approximately 25.08 km to the northwest, reaching Taiyuan Town, Hengyang County (112°26′ E, 27°05′ N). By the 2090s, it then turns to migrate approximately 76.13 km to the southwest, finally located at Fengqiping Township, Qidong County (111°41′ E, 27°00′ N). (3) Under the SSP585 scenario, by the 2050s, the centroid shifts approximately 140.13 km to the northwest, reaching Baomianqian Township, Shaodong County, Shaoyang City (112°04′ E, 27°03′ N). By the 2070s, it migrates approximately 15.36 km to the northeast, arriving at Da'an Township, Hengyang County (112°12′ E, 27°08′ N). By the 2090s, it then shifts approximately 4.90 km to the southwest, ultimately positioning at Jinlan Town, Hengyang County (112°09′ E, 27°07′ N). While most centroid shifts exceeded 10 km (range: 4.90–140.13 km), the smallest shift (SSP585‐2090s, 4.90 km) falls within the spatial resolution of the environmental layers (~1 km) and should be interpreted as a near‐stationary condition rather than evidence of directional migration.

## 4 Discussion

### 4.1 Parameter Optimization and Performance of the MaxEnt Model for *D. calycinum*

The parameter optimization implemented in this study substantiates research hypothesis H1, confirming that systematic parameter tuning significantly improves model complexity management and prediction reliability. Aligning with previous research indicating that default MaxEnt parameters frequently induce overfitting (Radosavljevic and Anderson 2014; Warren and Seifert 2011), our analysis revealed a substantially elevated Delta.AICc value of 50.42 under default parameters (RM = 1, FC = LQHP), demonstrating clear overfitting. Through a comprehensive grid search using the ENMeval package and AICc‐based model selection, we found that an optimally regularized model with a simplified feature set (higher RM and HPT features) not only eliminated the overfitting issue seen with default settings (which produced a much higher Delta.AICc) but also retained strong predictive accuracy. This underscores the necessity of parameter optimization for achieving a robust trade‐off between model fit and generalizability in species distribution modeling. This optimization strategy effectively controlled overfitting while enhancing model generalizability and robustness, adhering to the fundamental principle of balancing complexity and fit in model selection (Kass et al. 2021; Shabani et al. 2016). The simplified feature combination (HPT) was used, aligning with the feature reduction strategy recommended by Phillips and Dudík (2008) to enhance transferability and the practical optimization framework of Yang et al. (2024) for modeling medicinal plant distributions. In contrast, studies employing default parameters (Huang et al. 2018; Yi et al. 2016) likely contain prediction biases, highlighting the methodological advantage of our systematic optimization approach in ensuring prediction reliability and establishing a robust foundation for distribution modeling.

Beyond its immediate application to *D. calycinum* , our optimization methodology provides a transferable framework for distribution modeling of other subtropical medicinal plants. The optimized model enables more precise identification of species' ecological niche boundaries, offering a reliable basis for environmental factor analysis and habitat suitability delineation. Moreover, this approach can be effectively integrated into conservation planning and introduction area selection, supporting evidence‐based resource management strategies. Specifically, our optimized model can be directly applied to conservation priority zoning for medicinal plants as proposed in studies such as Gao et al. (2023) and Xia et al. (2022), thereby providing these research efforts with a validated tool to enhance zoning scientific rigor and practical applicability. Through its methodological sophistication, this study not only achieves high‐precision distribution modeling for *D. calycinum* but also establishes a reusable modeling framework for ecologically similar species (e.g., Daphniphyllum macropodum; Xiang et al. (2025)), thereby fulfilling its objective as a methodological paradigm with broad applicability in species distribution modeling.

### 4.2 Key Environmental Drivers and Ecological Response Thresholds for *D. calycinum*

The identification of annual mean temperature, dry‐season precipitation, and annual precipitation as the dominant factors underscores the overriding importance of water‐energy balance for *D. calycinum* , confirming hypothesis H2. Their cumulative contribution far exceeded that of other variable categories, collectively establishing climate as the primary determinant of the species' geographic range. This aligns with the expected physiological constraints on subtropical evergreen broadleaved trees (Chen et al. 2011; De Frenne et al. 2021). Specifically, the high importance of dry‐season precipitation corroborates the emphasis on moisture limitations during the driest period in research on *Quercus gilva* (Shi et al. 2024), another broadleaved evergreen tree inhabiting similar subtropical regions of China. This comparison is justified by their shared growth form and overlapping ranges, not by local co‐occurrence. The response curves further revealed nonlinear ecological responses with distinct thresholds (Section 3.2), indicating that both insufficient and excessive rainfall limit survival. Compared to earlier studies (Huang et al. 2018; Wang et al. 2025), our optimized modeling approach with rigorous variable screening more accurately characterizes these complex nonlinear relationships while effectively avoiding potential misinterpretations from variable collinearity (Dormann et al. 2013). Together, these findings support hypothesis H2 by demonstrating that climatic factors, particularly temperature and dry‐season precipitation, govern the distribution of *D. calycinum* through nonlinear responses with distinct ecological thresholds.

The precise quantification of key ecological thresholds carries significant practical implications. First, these thresholds help delineate priority conservation zones concentrated in Guangxi, Guangdong, and Jiangxi provinces, rather than broadly applying climatic ranges across southern China. Second, they guide cultivation site selection by identifying areas that meet the species' requirements while avoiding regions where annual precipitation exceeds the upper limit or dry‐season precipitation falls below the minimum. Third, the projected northwestward shift of suitable habitats suggests that central‐southern Hunan and northern Jiangxi should be prioritized for assisted migration. These findings translate ecological thresholds into spatially explicit guidance for conservation and introduction planning, and can be integrated into broader frameworks for threatened medicinal plants in China (Gao et al. 2023; Xia et al. 2022).

Regarding the low contribution of non‐climatic factors (topography, human footprint, vegetation < 3%), we offer two complementary explanations. First, this result likely reflects the species' inherent ecological specialization. *D. calycinum* , as a subtropical evergreen broadleaf tree, may have its distribution primarily constrained by broad‐scale climatic regimes rather than by local topographic or anthropogenic factors, a pattern observed in other subtropical species (Shi et al. 2024; Xiang et al. 2025). The low contribution of the Human Footprint Index (1.3%) suggests that under current conditions, *D. calycinum* primarily occupies regions with relatively low anthropogenic pressure, rather than human‐dominated landscapes (Lu et al. 2020; Williams et al. 2020). Second, methodological factors may have underestimated their importance. The coarse spatial resolution (~1 km) of both topographic and human footprint data may fail to capture fine‐scale heterogeneity, such as local habitat fragmentation, harvesting pressure, or microtopographic refugia (Mu et al. 2022). Moreover, our current Human Footprint Index reflects contemporary conditions, whereas future land‐use changes were not incorporated into our climate‐only projections, which is a common but recognized limitation (Powers and Jetz 2019). Consequently, our estimates of future suitable areas may represent an optimistic scenario that assumes no increase in anthropogenic barriers (Newbold 2018). Future studies should integrate dynamic land‐use scenarios and higher‐resolution edaphic and microtopographic variables (Franklin 2023; Lembrechts et al. 2019) to better define species‐specific habitat suitability (Jung et al. 2021; Trisos et al. 2020). Despite these limitations, this study establishes baseline ecological thresholds for more grounded conservation planning of *D. calycinum* .

### 4.3 Projected Spatio‐Temporal Shifts in Habitat Suitability for *D. calycinum* Under Climate Change

The consistent expansion of suitable habitat across all future scenarios suggests that future climate conditions may broadly favor this species, particularly under the high‐emission SSP585 pathway. The total suitable area reaches its maximum by the 2050s under SSP585, with an expansion of nearly 47% relative to the baseline period. Spatially, high‐suitability areas progressively shift northwestward from their current concentration in coastal South China toward inland regions, accompanied by a marked northwestward migration of the distribution centroid. This systematic centroid shift toward higher latitudes and altitudes aligns with macroecological patterns of climate‐driven range reorganization (Soroye et al. 2020) and corroborates findings that climate change facilitates range expansions in some species (Warren et al. 2018), directly validating component H3 of our research hypothesis.

Ecologically, these distribution patterns reflect integrated effects of multiple processes. Temperature increase serves as the primary driver of northwestward centroid migration (Pecl et al. 2017), while precipitation variations modulate expansion magnitude across scenarios, refining theoretical understanding of interactive climatic effects on species distributions (Elith et al. 2011). The observed spatial heterogeneity in habitat changes corresponds with frameworks positing species responses to climate change through integrated effects of evolutionary history and contemporary adaptive potential (Lavergne et al. 2010). This indicates that distributional changes in *D. calycinum* are constrained by its fundamental niche yet may be facilitated by phenotypic plasticity, enabling colonization of new geographic spaces.

In contrast to many sympatric subtropical tree species predicted to experience range contraction under climate change, *D. calycinum* shows consistent habitat expansion. For example, *Quercus gilva* is projected to lose 39.4%–77.1% of its suitable habitat due to its narrow thermal niche and strong dry‐season moisture dependence (Shi et al. 2024). Similarly, *Fagus longipetiolata* and *Cercidiphyllum japonicum* are expected to face significant range contraction as rapid warming exceeds their physiological limits (Jiang et al. 2025; Yin and Zhou 2015). These contrasting trajectories can be explained by the niche characteristics of *D. calycinum* identified in Section 4.2. First, it possesses a broad thermal tolerance range (14.39°C–26.17°C) and high upper precipitation threshold (~2265 mm), enabling tolerance to warmer conditions. Second, its non‐linear response to dry‐season precipitation shows no sharp decline beyond ~80 mm, contrasting with the high moisture sensitivity of *Quercus gilva*. Third, the low contribution of non‐climatic factors (< 3%) suggests it functions as a climate‐generalist with less microhabitat specialization. Consequently, future warming may open new suitable windows at higher latitudes without exceeding its physiological limits. These findings highlight that fundamental niche breadth is a key determinant of species' vulnerability or opportunism under climate change, underscoring the novelty of our response curve‐based assessment.

Compared with the established consensus on climate‐driven poleward range shifts Lenoir and Svenning (2015), our study not only confirms this general trend but also quantitatively demonstrates significant variation in habitat expansion magnitude across different emission scenarios. Methodologically, our parameter optimization effectively addressed overfitting issues prevalent in earlier studies (Wang, Xie, et al. 2023), as evidenced by the reduction in Delta.AICc from 50.42 (default settings) to 0 (optimized model), thereby enhancing prediction reliability. Furthermore, compared with research on congeneric *D. macropodum* (Xiang et al. 2025), our study reveals fluctuating growth patterns of suitable areas under SSP370 and SSP585 scenarios, reflecting greater tolerance to extreme climate events and providing new insights into climatic adaptation divergence among closely related species.

From a conservation perspective, these findings offer explicit guidance for sustainable resource utilization. Based on dynamic habitat predictions, we suggest considering northern Guangxi and northwestern Guangdong as priority zones for in situ conservation monitoring within a broader conservation network (Dinerstein et al. 2020). Emerging suitable areas in central‐southern Hunan and northern Jiangxi may be considered for experimental cultivation trials, contingent on further research into dispersal ecology and adaptive management frameworks. Centroid migration trajectories provide a scientific basis for constructing regional ecological corridor networks, recommending enhanced habitat connectivity conservation in critical ecological nodes like the Nanling Mountain and Wuyi Mountain (Mokany et al. 2022) to facilitate natural migration processes. In summary, the stark contrast in habitat changes across emission scenarios underscores the decisive influence of socioeconomic pathways on biodiversity (Powers and Jetz 2019), providing robust ecological justification for integrating biodiversity conservation objectives into regional climate adaptation and mitigation policies. These projections assume unlimited dispersal and no future land‐use change; therefore, realized colonization may be lower.

### 4.4 Study Limitations and Scope of Inference

#### 4.4.1 Methodological Limitations

Several methodological limitations should be acknowledged. First, reliance on a single climate model (BCC‐CSM2‐MR) introduces uncertainty in future projections; although this model performs well for East Asian climates, it cannot fully capture the range of possible climate futures. Future studies should employ multi‐GCM ensembles (e.g., BCC‐CSM2‐MR, CanESM5, MIROC6) to quantify inter‐model variability, preferably with downscaled data to identify microclimatic refugia. Second, key environmental predictors including elevation, soil properties, and land‐use factors were omitted due to lack of high‐resolution datasets. Future research should integrate these variables to refine habitat assessments. Third, our model validation used random cross‐validation instead of spatially structured partitioning (e.g., block or checkerboard), which may overestimate performance due to spatial autocorrelation. Future studies should adopt spatially explicit cross‐validation (e.g., via ENMeval) for more robust assessment. Fourth, although we justified our use of the 10‐percentile training presence threshold, a formal sensitivity analysis comparing alternative thresholds was not conducted. Future studies should systematically compare multiple thresholds to assess the robustness of area estimates and expansion trends. Fifth, we did not present uncertainty estimates (e.g., standard deviation maps) for future projections. Future studies should report such metrics to improve transparency, particularly at range margins where model confidence is lower.

#### 4.4.2 Dispersal Assumptions and Optimistic Bias

A critical assumption of our MaxEnt model is unlimited dispersal, implying that all climatically suitable areas are potentially accessible to the species. In reality, *D. calycinum* may face limited seed dispersal and habitat fragmentation due to land‐use change and natural barriers. These factors could constrain range expansion, as isolated suitable habitats may remain uncolonized. Thus, our projections likely represent an optimistic upper bound of potential suitable areas rather than deterministic forecasts of realized colonization. Future studies should integrate dispersal constraints (e.g., dispersal kernels or least‐cost models) and dynamic land‐use scenarios for more realistic estimates.

#### 4.4.3 Geographic and Temporal Scope of Inference

The conclusions of this study are subject to specific geographic and temporal boundaries. First, the predictions are geographically restricted to China, where the species' natural distribution occurs, and should not be directly extrapolated to other regions without additional validation. Second, the projections are valid for the time horizon up to the 2090s under the three SSP scenarios considered (SSP126, SSP370, SSP585); predictions beyond this period or under unexamined scenarios remain uncertain. Third, as noted in Section 4.4.2, our assumption of unlimited dispersal means that our estimates represent an optimistic upper bound of potential suitable areas, rather than realized colonization. Therefore, while our findings provide robust spatial guidance for conservation prioritization and cultivation planning within China, they should be interpreted as potential climatic suitability contingent on dispersal opportunities and the absence of novel biotic or anthropogenic barriers. Future applications of this model should consider local ground‐truthing and adaptive management strategies.

## 5 Conclusion

This study demonstrates that climatic factors, particularly annual mean temperature (Bio1) and dry season precipitation (Bio14), are the primary determinants of potential climatic suitability for *D. calycinum* in China, with the optimized MaxEnt model achieving high predictive accuracy (AUC = 0.959, TSS = 0.800). Using an optimized MaxEnt model (RM = 2.0, FC = HPT), we project a general poleward range shift and net expansion of climatically suitable habitat under future scenarios. However, this net expansion coexists with localized habitat loss and, under certain scenarios, a declining proportion of highly suitable areas, suggesting that *D. calycinum* may act as a climate opportunist in newly suitable areas while facing pressure within its current range core. Based on these findings, we recommend proactive monitoring of stable core habitats (Guangxi, Guangdong, Jiangxi), experimental cultivation trials in emerging suitable areas (central southern Hunan and northern Jiangxi) contingent on empirical validation of dispersal ecology, and enhanced habitat connectivity conservation in the Nanling and Wuyi mountains. Our projections represent potential climatic suitability rather than deterministic forecasts, and future research should integrate dispersal constraints, dynamic land use scenarios, and multi‐model ensembles to refine predictions.

## Author Contributions

**Yangzhou Xiang:** conceptualization (lead), data curation (lead), formal analysis (lead), funding acquisition (lead), methodology (lead), writing – original draft (lead). **Suhang Li:** conceptualization (supporting), data curation (supporting), validation (lead), writing – review and editing (supporting). **Qiong Yang:** conceptualization (lead), data curation (supporting), formal analysis (supporting), investigation (lead), software (supporting), writing – review and editing (supporting). **Ying Liu:** conceptualization (supporting), data curation (lead), formal analysis (supporting), investigation (equal), methodology (lead), writing – review and editing (supporting). **Bin Yao:** conceptualization (supporting), funding acquisition (lead), methodology (supporting), project administration (lead), resources (lead), writing – review and editing (supporting). **Huilin Dong:** conceptualization (lead), funding acquisition (lead), project administration (lead), resources (lead), supervision (lead), writing – original draft (supporting). **Yuan Li:** conceptualization (lead), investigation (supporting), writing – review and editing (lead).

## Funding

This work was supported by the Fundamental Research Funds for the Guizhou Provincial Science and Technology Projects (QKHJC‐ZK [2022] YB335) and the Guizhou Education University Scientific Research Fund Project (2024YB002; 2024BSKQ003).

## Conflicts of Interest

The authors declare no conflicts of interest.

## Supporting information


**Table S1:** Twenty‐four environmental variables used in this study.

## Data Availability

Species location records and environmental variables have been uploaded to an open data repository via Figshare (https://doi.org/10.6084/m9.figshare.30828209).
