# Supplementary material for: MaxEnt Projections of Climate‐Driven Distribution Shifts for Daphniphyllum calycinum in China
Source: Ecol Evol. 2026 Jul 31;16(8):e74138. doi: 10.1002/ece3.74138 (PMC13425653; doi:10.1002/ece3.74138)
Supplement: Supplementary file 1 — Table S1: Twenty‐four environmental variables used in this study. [file ECE3-16-e74138-s001.docx]

Supplementary materials

**MaxEnt Projections of Climate-Driven Distribution Shifts for *Daphniphyllum calycinum* in China**

Yangzhou Xiang ^1^, Suhang Li ^1^, Qiong Yang ^1^, Ying Liu ^2^, Bin Yao ^3, *^, Huilin Dong ^1, *^, Yuan Li ^4,*^

^1^ School of Geography and Resources, Guizhou Education University, Guiyang 550018, China

^2^ School of Biological Sciences, Guizhou Education University, Guiyang 550018, China

^3^ State Key Laboratory of Tree Genetics and Breeding, Institute of Ecology Conservation and Restoration, Chinese Academy of Forestry, Beijing, China
^4^ Grasslands and Sustainable Farming, Production Systems Unit, Natural Resources Institute Finland, Halolantie 31 A, 71750, Maaninka, Finland

*Correspondence to: acmn21@caf.ac.cn (B.Y.); dzjxhd@163.com (H. D.); yuan.li@luke.fi (Y. Li).

Table S1. Twenty-four environmental variables used in this study.

| Category | Abbreviation | Environmental variables | Units | Range | Percent contribution |
| --- | --- | --- | --- | --- | --- |
| Bioclimatic | Bio1 | Annual mean temperature | °C | 14.45－25.85 | 0.2 |
|  | Bio2 | Mean diurnal range (Mean of monthly) | °C | 5.94－12.08 | 0.4 |
|  | Bio3 | Isothermality (Bio2/Bio7) (× 100) |  | 23.50－52.52 | 1.3 |
|  | Bio4 | Standard deviation of temperature seasonality |  | 279.83－869.64 | 0.5 |
|  | Bio5 | Max temperature of warmest month | °C | 24.07－34.29 | 0.1 |
|  | Bio6 | Min temperature of coldest month | °C | 0.26－17.77 | 29.6 |
|  | Bio7 | Temperature annual range (Bio5-Bio6) | °C | 14.41－32.38 | 0.4 |
|  | Bio8 | Mean temperature of wettest quarter | °C | 18.19－28.85 | 0.1 |
|  | Bio9 | Mean temperature of driest quarter | °C | 4.97－22.85 | 3.7 |
|  | Bio10 | Mean temperature of warmest quarter | °C | 19.03－28.98 | 0.1 |
|  | Bio11 | Mean temperature of coldest quarter | °C | 4.89－21.99 | 1.2 |
|  | Bio12 | Annual precipitation | mm | 909.00－2906.00 | 33.1 |
|  | Bio13 | Precipitation of wettest month | mm | 181.00－507.00 | 0.2 |
|  | Bio14 | Precipitation of driest month | mm | 12.00－166.00 | 0.1 |
|  | Bio15 | Variation of precipitation seasonality |  | 23.83－89.86 | 0.5 |
|  | Bio16 | Precipitation of wettest quarter | mm | 482.00－1428.00 | 0.0 |
|  | Bio17 | Precipitation of driest quarter | mm | 41.00－534.00 | 0.1 |
|  | Bio18 | Precipitation of warmest quarter | mm | 358.00－1428.00 | 0.0 |
|  | Bio19 | Precipitation of coldest quarter | mm | 41.00－558.00 | 2.9 |
| Topographic | Altitude | Altitude | m | 0.00－2067.00 | 17.1 |
|  | Aspect | Aspect | ° | 0.00－358.25 | 0.4 |
|  | Slope | Slope | ° | 0.03－5.22 | 0.4 |
| Human | HFI | Human footprint index |  | 3.77－50.00 | 7.3 |
| Vegetation | NDVI | Normalized difference vegetation index |  | 0.14－0.82 | 0.2 |
